# Impact of COVID-19 on Life Experiences of Essential Workers Attending a Dental Testing Facility

**DOI:** 10.1177/2380084420962399

**Published:** 2020-09-22

**Authors:** M. Fontana, L. McCauley, M. Fitzgerald, G.J. Eckert, E. Yanca, R. Eber

**Affiliations:** 1 Department of Cariology, Restorative Sciences and Endodontics, School of Dentistry, University of Michigan, Ann Arbor, MI, USA; 2 Department of Periodontics and Oral Medicine, School of Dentistry, University of Michigan, Ann Arbor, MI, USA; 3 Department of Biostatistics, School of Medicine, and Richard M. Fairbanks School of Public Health, Indiana University, Indianapolis, IN, USA

**Keywords:** dentist, health care, stress, anxiety, pandemic, life quality

## Abstract

COVID-19 poses a threat to health and well-being.

**Objectives:**

1) To compare the impact of COVID-19 on the life experiences of essential workers attending a COVID-19 antibody testing clinic at a dental school. 2) To compare responses of dental, non–dental health care, and non–health care essential workers. 3) To assess acceptability/satisfaction of testing done in a dental setting.

**Method:**

A total of 984 participants completed a self-administered online questionnaire.

**Results:**

Over 90% were healthy (i.e., not in a high-risk health-related group for COVID-19), did not have COVID-19 symptoms within 30 d, and always/frequently engaged in preventive measures. Fifty-eight percent thought that they had a 0% to 25% chance of having immunity/antibodies to COVID-19. Non–dental health care workers thought that their chance was significantly higher (P < 0.05) than others. Over 70% were sometimes, frequently, or always worried about their friends and loved ones getting COVID-19 and of resulting financial problems. Dental workers were significantly less afraid than non–dental health care and non–health care providers. For all groups, more than half of the respondents stated that the pandemic had a negative (somewhat worse or worse) impact on daily life (59%), interactions with others (65%), stress levels (66%), and enjoyment of life (56%). There were significant differences among all 3 groups regarding the percentage of individuals with a negative impact on job security (dental, 47%; non–dental health care, 34%; non–health care, 31%). However, more than half of the respondents stated that the pandemic had a positive impact (same, somewhat better, or much better) on caring about one another, self-care, and exercise. Knowing the results of an antibody test would decrease the level of stress and anxiety in 67% of respondents. Over 80% found a COVID-19 test received in a dental setting acceptable, were “definitely” satisfied, and would “definitely” recommend it to a friend, family, or coworker.

**Conclusions:**

These findings support that dental workers are as vulnerable as other essential workers to threats and psychological impacts of COVID-19. They also support the acceptability and satisfaction of testing for a pandemic done in a dental setting.

**Knowledge Transfer Statement:**

The results of this study highlight the impact that pandemics such as COVID-19 can have on life experiences of essential workers, including dentists. It also highlights a role that dentistry can play within the broader health care system, during and beyond the current pandemic, to help with surveillance efforts of community health. Testing may also help alleviate stress and anxiety associated with these pandemics.

## Introduction

The 2019 novel coronavirus disease (COVID-19), first identified in Wuhan, China (Centers for Disease Control and Prevention [CDC] 2020a), is caused by the severe acute respiratory syndrome coronavirus 2 (SARS-CoV-2), which belongs to the Coronaviridae, a family of viruses that possess a positive-sense single-stranded RNA genome (Tu et al. 2020). On March 11, 2020, COVID-19 was declared a pandemic and on March 13 a national emergency in the United States (CDC 2020a).

SARS-CoV-2 is thought to spread mainly from person to person through respiratory droplets. Fecal-oral spread may also be possible (CDC 2020a). Infected individuals can be contagious before the onset of symptoms (Tu et al. 2020), and some remain relatively asymptomatic yet contagious (Guan et al. 2020). The most frequent symptoms of COVID-19 include fever, chills, cough, shortness of breath, fatigue, and muscle aches (C. Huang et al. 2020; Tu et al. 2020). In addition, some patients have sore throat, rhinorrhea, and headache a few days before fever onset. As the respiratory disease progresses, pneumonia can develop, exhibiting a broad spectrum of severity/progression. SARS-CoV-2 can also affect intestinal tissues, resulting in gastrointestinal symptoms (e.g., nausea, vomiting, diarrhea; Tu et al. 2020). In addition, hypogeusia and hyposmia have been reported as common neurologic manifestations (Mao et al. 2020).

Several types of tests can identify SARS-CoV-2 RNA, viral antigens, or antibodies from the host’s immune response. The most sensitive of these tests, the reverse transcription polymerase chain reaction test, generally requires a nasopharyngeal or oropharyngeal swab and takes several hours of processing in a laboratory. As resources for this test have been in short supply, it has been primarily limited in the United States to use in sick or presumptive-sick individuals and some selected at-risk groups. Rapid antigen tests are also under development but likely with the drawback of decreased accuracy. In addition, serologic tests are important in the management of infectious diseases, as they may aid in diagnosis, measuring protective antibody titers postvaccination and assessing seroprevalence of immunity in the population (Krammer and Simon 2020). Many antibody (IgG, IgM) point-of-care (POC) tests have been developed to detect the immune response to SARS-CoV-2. These tests are relatively simple and inexpensive, and they provide a response within 10 to 15 min. The recent COVID-19 Serology Studies Workshop (Lerner et al. 2020) concluded that more research is needed to determine if and to what extent a positive antibody test means that a person may be protected from reinfection with SARS-CoV-2. IgG antibodies appear in blood around 10 d after infection, when titers of the virus are decreasing, and thus cannot aid in early diagnosis but may help determine exposure to the virus. Levels of IgM antibodies are high in symptomatic and asymptomatic patients 5 d after onset of illness. It has been proposed that IgM POC assays followed by polymerase chain reaction for positive responses could enhance the detection sensitivity of these tests (Guo et al. 2020; Krammer and Simon 2020). These POC tests, once better understood and studied, could aid with community surveillance of the disease and could be deployed in many settings, including dental settings.

COVID-19 has caused huge threats to health and well-being, resulting in problems such as panic, anxiety, depression, posttraumatic stress disorders, suspiciousness, infodemia, and xenophobia (Jakovljevic et al. 2020). These might be associated with concerns about being affected by the virus or with the prevention measures and/or socioeconomic impact of the pandemic. In addition, certain population groups may be at additional psychological distress, including health care workers and other essential workers (Talevi et al. 2020; World Health Organization 2020), yet very little is known about the impact on life experiences of dental personnel, particularly in the United States.

As dentists are an important part of the health care team and can play an important interdisciplinary role in disease surveillance and triage, from May to June 2020 the University of Michigan School of Dentistry (UMSD) implemented a pilot-testing clinic for COVID-19 using a rapid POC IgG-IgM antibody test for SARS-CoV-2. The test was offered to essential personnel. The objectives of this study were to 1) compare the impact of COVID-19 on the life experiences of essential personnel attending the COVID-19 antibody testing clinic at the UMSD, 2) compare responses of dental (D) personnel with non–dental health care (ND) and non–health care (NH) essential workers, and 3) assess the acceptability of performing antibody tests in dental settings.

## Methods

This descriptive cross-sectional study was deemed exempt by the University of Michigan Institutional Review Board (HUM00180555), and reporting follows the STROBE guidelines.

Essential workers, associated or not with the University of Michigan, who attended the UMSD’s COVID-19 testing clinic were eligible to participate in this study. To be tested, individuals were prescreened for COVID-19 symptoms, recent exposure to someone who was COVID-19 positive, and travel outside the country within 2 wk, and their temperature was taken prior to entry in the dental building. Only asymptomatic participants were tested. Prior to testing, they were informed of the need for more evidence to understand the value of POC tests in the current pandemic, and they were given details about the test used (lateral flow kit with finger stick, Duo IgM/IgG [SD Biosensor]; not Food and Drug Administration approved but marketed through initial rules for use while waiting for emergency use authorization). The University of Michigan Department of Pathology tested 120 kits using known serum from patients in the university medical system who were COVID-19 positive and negative, and it found a 97.1% specificity and 97.3% sensitivity. Through collaboration with the T4T group (Testing for Tomorrow; including Henry Schein, Inc., University of Michigan, New York University, University of Pennsylvania, Temple University, and University of California San Francisco), the UMSD obtained 1,000 test kits to use for the dental implementation project: 3 were used for training and 997 were available for use; thus, 997 was the study’s target sample size.

Individuals were invited to participate in this research study by a trained study dentist. To be included, they needed to be ≥18 y old. Exclusion criteria included inability to comply with study protocol requirements and non-English reading/speaking.

After providing electronic consent, study participants completed a 58-item self-administered online survey (Qualtrics) while they waited for the antibody test results. Questions were informed, when available, by those starting to be posted in mid-April in 2 online COVID-19 toolkits (National Institutes of Health 2020; PhenX Toolkit 2020).

The following variables were assessed: 1) demographics (age, sex, race, ethnicity); 2) employment and changes since the Michigan’s stay-at-home order; 3) health as related to signs, symptoms, and risk of COVID-19; 4) perception of the likelihood of having antibodies to the virus; 5) frequency of preventive habits in daily life (e.g., social distancing, wearing mask in public); 6) perceived impact and threat of COVID-19 on life experiences and well-being (finances, health, stress, etc.); 7) expected impact of antibody test results on level of stress and anxiety; 8) self-reported results of the antibody test; and 9) acceptability/satisfaction with the testing protocol.

For analyses, data were exported from the website as an SPSS data file (SPSS version 22.0; IBM Corp.). Descriptive statistics such as frequencies/percentages and means/standard deviations were computed to provide an overview of the responses. Chi-square and Kruskal-Wallis tests were used to compare responses among types of provider (D, ND, and NH) for nominal categorical variables and ordinal or continuous variables, respectively. When the overall test result was significant, pairwise chi-square tests or Dwass-Steel-Critchlow-Fligner multiple-comparisons analysis (based on pairwise Wilcoxon rank sum tests) was performed. A 5% significance level was used for all tests. Statistical analyses were performed with SAS version 9.4 (SAS Institute Inc.).

## Results

Of the 997 individuals approached, 984 agreed to participate, completed the survey, and identified themselves as part of 1 of 3 groups: dental (D), non–dental health care (ND), or non–health care (NH) essential workers. Six did not complete the work-related questions and thus were excluded from the analyses. The mean ± SD age of respondents was 41.8 ± 13.4 y (range, 18 to 78 y). Although of similar age, the NH group was significantly (*P* < 0.05) older (*n* = 265, 27%; 43.9 ± 13.8 y; range, 18 to 80 y) than the D group (*n* = 416, 42%; 41.1 ± 14.3 y; range, 18 to 78 y) and ND group (*n* = 303, 31%; 40.8 ± 11.6 y; range, 19 to 69 y). Among the groups, there were no significant differences for sex (37% male, 63% female), race (81% White, 4% Black, 12% Asian, <1% Hawaiian or Pacific islander, <1% American Indian or Alaskan native, 2% multiracial), and ethnicity (94% non-Hispanic or Latino, 6% Hispanic or Latino). Work-related information is presented in Table 1. Dental workers were significantly more likely than the others to use personal protective equipment at work prior to COVID-19 and be unemployed/no longer working after the Michigan stay-at-home order. After the stay-at-home order, ND workers were significantly more likely than the other groups to continue working, be working at a different location, and have direct contact with people/patients likely to have COVID-19, whereas NH workers were significantly more likely than the others to be working from home and reassigned to different duties.

**Table 1.** Work Characteristics prior to and after COVID-19 Stay-at-Home Order in Michigan.

	Respondents, *n* (%)				*P* Value			
Question: Response	All	D	ND	NH	Overall	D vs. ND	D vs. NH	ND vs. NH
Did you routinely use personal protective equipment (mask, gloves, gowns, etc.) at your work prior to COVID-19?								
Yes	715 (73)	393 (95)	221 (73)	101 (38)	<0.001	<0.001	<0.001	<0.001
No	267 (27)	22 (5)	82 (27)	163 (62)				
Missing	2	1		1				
Since Michigan’s shelter-in-place order, have there been any changes to your job status, duties, or location?								
Yes	499 (51)	234 (56)	116 (38)	149 (56)	<0.001	<0.001	0.995	<0.001
No	485 (49)	182 (44)	187 (62)	116 (44)				
Missing	0	0	0	0				
Since Michigan’s shelter-in-place order that started on March 24, 2020, which of the following describes changes to your job? (Select all that apply)								
No longer working / unemployed	93 (19)	83 (35)	1 (1)	9 (6)	<0.001	<0.001	<0.001	0.028
Working from home	261 (52)	82 (35)	53 (46)	126 (85)	<0.001	0.054	<0.001	<0.001
Working at a different location, not home	27 (5)	8 (3)	16 (14)	3 (2)	<0.001	<0.001	0.422	<0.001
Working, but reassigned to different duties	77 (15)	21 (9)	46 (40)	10 (7)	<0.001	<0.001	0.429	<0.001
Working, but reassigned to less hours per week	131 (26)	76 (32)	35 (30)	20 (13)	<0.001	0.663	<0.001	0.001
Started working / came out of retirement	8 (2)	5 (2)	1 (1)	2 (1)	0.641			
During Michigan’s shelter-in-place order that started on March 24, 2020, were you required to continue working because your job was considered essential?								
Yes	543 (55)	158 (38)	281 (93)	104 (39)	<0.001	<0.001	<0.001	<0.001
No (but working from home)	233 (24)	84 (20)	17 (6)	132 (50)				
No (I am not working)	208 (21)	174 (42)	5 (2)	29 (11)				
Are you required to have direct contact with people/patients who are likely to have COVID-19 (i.e., people who have tested positive or with suspected symptoms)?								
Yes	236 (43)	24 (15)	203 (72)	9 (9)	<0.001	<0.001	0.119	<0.001
No	307 (57)	134 (85)	78 (28)	95 (91)				
Missing	441	258	22	161				

D, dental workers; ND, non–dental health care workers; NH, non–health care workers.

Information about participants’ health and risk of COVID-19 and their perception of the likelihood of having immunity to the virus is presented in Table 2. Very few participants reported having COVID-19 symptoms within the previous 30 d, with no significant differences among the groups. Very few participants also reported being part of a health-related high-risk group for COVID-19. However, a significantly higher number of ND workers versus the other 2 groups thought (or were unsure) that they had been infected with COVID-19, had been tested, and had been in close contact with someone suspected or confirmed to have had COVID-19. A significantly lower number of D participants than the others had sought medical care because they suspected that they had COVID-19. Yet, out of all the study participants, only 1 person (a dentist) reported having tested positive for the virus. Also, when compared with the other groups, the ND group reported significantly more frequent exposure to the general public since the beginning of the pandemic. Not surprising, although significantly different between themselves, the D and ND groups had significantly more frequent contact with patients/clinical care than the NH group since the beginning of the pandemic. Overall, 58% of participants thought that they had a ≤25% chance of having immunity/antibodies to COVID-19, with an additional 30% thinking that their chance was between 26% and 50%. Only 11% thought that their chance was >51%. ND workers believed that their chance was significantly higher than the other groups, with the D and NH groups being not significantly different.

**Table 2.** Participants Health and Risk of COVID-19 and Perception of Likelihood of Having Antibodies to the Virus.

	Respondents, *n* (%)				*P* Value			
Question: Response	All	D	ND	NH	Overall	D vs. ND	D vs. NH	ND vs. NH
What has been your exposure to the general public since the beginning of the pandemic?								
None or less than once every 2 wk	45 (8)	12 (8)	18 (6)	15 (14)	0.001	0.003	0.993	0.023
Once every 2 wk	39 (7)	12 (8)	18 (6)	9 (9)				
1 to 2 times per week	160 (29)	57 (36)	73 (26)	30 (29)				
3 to 4 times per week	127 (23)	40 (25)	66 (23)	21 (20)				
5 to 6 times per week	80 (15)	26 (16)	42 (15)	12 (12)				
Every day	92 (17)	11 (7)	64 (23)	17 (16)				
Missing	441	258	22	161				
Had you been out of the country during January to March 2020?								
Yes	132 (13)	70 (17)	44 (15)	18 (7)	0.001	0.403	<0.001	0.003
No	852 (87)	346 (83)	259 (85)	247 (93)				
If you are working in health care, what has been your exposure to patients/clinical care since the beginning of the pandemic?								
N/A (i.e., do not work in health care)	62 (11)	4 (3)	1 (<1)	57 (56)	<0.001	<0.001	<0.001	<0.001
None or less than once every 2 wk	78 (14)	27 (17)	31 (11)	20 (20)				
Once every 2 wk	37 (7)	13 (8)	21 (7)	3 (3)				
1 to 2 times per week	105 (19)	59 (37)	42 (15)	4 (4)				
3 to 4 times per week	121 (22)	34 (22)	79 (28)	8 (8)				
5 to 6 times per week	65 (12)	16 (10)	46 (16)	3 (3)				
Every day	72 (13)	5 (3)	61 (22)	6 (6)				
Missing	444	258	22	164				
Have you had any of the following symptoms in the last 30 d? Fever >100 ° F								
Yes	10 (1)	3 (1)	4 (1)	3 (1)	0.714			
No	974 (99)	413 (99)	299 (99)	262 (99)				
Have you had any of the following symptoms in the last 30 d? Cough								
Yes	27 (3)	9 (2)	12 (4)	6 (2)	0.298			
No	956 (97)	406 (98)	291 (96)	259 (98)				
Missing	1	1						
Have you had any of the following symptoms in the last 30 d? Difficulty breathing								
Yes	13 (1)	5 (1)	7 (2)	1 (<1)	0.127			
No	970 (99)	410 (99)	296 (98)	264 (100)				
Missing	1	1						
Have you had any of the following symptoms in the last 30 d? Fatigue or tiredness								
Yes	60 (6)	17 (4)	25 (8)	18 (7)	0.062			
No	923 (94)	398 (96)	278 (92)	247 (93)				
Missing	1	1						
Have you had any of the following symptoms in the last 30 d? Loss of sense of smell or taste								
Yes	9 (1)	3 (1)	4 (1)	2 (1)	0.673			
No	974 (99)	412 (99)	299 (99)	263 (99)				
Missing	1	1						
Do you think you’ve been infected with COVID-19?								
Yes	61 (6)	14 (3)	32 (11)	15 (6)	<0.001	<0.001	0.065	<0.001
No	564 (57)	282 (68)	124 (41)	158 (60)				
Unsure	359 (36)	120 (29)	147 (49)	92 (35)				
Have you ever sought medical care because you suspected you may have been infected with COVID-19?								
Yes	62 (6)	15 (4)	28 (9)	19 (7)	0.007	0.002	0.037	0.371
No	922 (94)	401 (96)	275 (91)	246 (93)				
Have you ever been tested for COVID-19 to know if you had the virus (not counting today)?								
I have been tested	71 (7)	24 (6)	35 (12)	12 (5)	0.002	0.005	0.480	0.002
I have not been tested	913 (93)	392 (94)	268 (88)	253 (95)				
What were the results?								
I tested positive (I had COVID-19)	1 (1)	1 (4)			0.275			
I tested negative (I did not have COVID-19)	67 (94)	23 (96)	32 (91)	12 (100)				
I do not know/remember the result	3 (4)		3 (9)					
Missing	913	392	268	253				
Whether or not you have been tested for COVID-19, has a doctor or another health care professional ever diagnosed you as having or probably having COVID-19?								
Yes	11 (1)	4 (1)	4 (1)	3 (1)	0.708			
No	952 (97)	403 (97)	295 (97)	254 (96)				
Unsure	21 (2)	9 (2)	4 (1)	8 (3)				
How severe was your illness?								
Mild symptoms, cough, no difficulty breathing	5 (45)	2 (50)	1 (25)	2 (67)	0.566			
Moderate, some difficulty breathing, not requiring hospitalization	6 (55)	2 (50)	3 (75)	1 (33)				
Missing	973	412	299	262				
Have you ever been tested to determine if you are antibody positive (immune) to COVID-19?								
Yes	33 (3)	15 (4)	13 (4)	5 (2)	0.402			
No	950 (97)	400 (96)	290 (96)	260 (98)				
Unsure	1 (<1)	1 (<1)						
What do you think your chance is of having immunity/antibodies to COVID-19? Please provide your best guess, even if you are unsure.								
0% to 25%	574 (58)	270 (65)	143 (47)	161 (61)	<0.001	<0.001	0.387	0.004
26% to 50%	299 (30)	114 (27)	112 (37)	73 (28)				
51% to 75%	86 (9)	29 (7)	36 (12)	21 (8)				
76% to 100%	24 (2)	3 (1)	12 (4)	9 (3)				
Missing	1			1				
Have you ever been in physical contact with anyone that has developed infection that is suspected or confirmed to be COVID-19?								
Yes	217 (22)	45 (11)	147 (49)	25 (9)	<0.001	<0.001	0.555	<0.001
No	766 (78)	370 (89)	156 (51)	240 (91)				
Missing	1	1						
How long ago was the exposure?								
1-2 wk ago	21 (10)	2 (4)	18 (12)	1 (4)	<0.001	<0.001	0.992	0.005
3 wk ago	47 (22)	2 (4)	44 (30)	1 (4)				
>3 wk ago	149 (69)	41 (91)	85 (58)	23 (92)				
Missing	767	371	156	240				
Are you in any of the following high-risk categories for severe COVID-19 infection? Live in a nursing home or long-term care facility								
Yes	2 (<1)		2 (1)		0.106			
No	981 (100)	415 (100)	301 (99)	265 (100)				
Missing	1	1						
Are you in any of the following high-risk categories for severe COVID-19 infection? Chronic lung disease or moderate to severe asthma								
Yes	47 (5)	13 (3)	13 (4)	21 (8)	0.015	0.412	0.005	0.069
No	936 (95)	402 (97)	290 (96)	244 (92)				
Missing	1	1						
Are you in any of the following high-risk categories for severe COVID-19 infection? Serious cardiovascular disease								
Yes	11 (1)	3 (1)	3 (1)	5 (2)	0.360			
No	972 (99)	412 (99)	300 (99)	260 (98)				
Missing	1	1						
Are you in any of the following high-risk categories for severe COVID-19 infection? Conditions that can cause a person to be immunocompromised (e.g., cancer treatment, smoking, bone marrow or organ transplantation, immune deficiencies, poorly controlled HIV or AIDS, and prolonged use of corticosteroids and other immune weakening medications)								
Yes	42 (4)	11 (3)	12 (4)	19 (7)	0.017	0.328	0.005	0.093
No	940 (96)	403 (97)	291 (96)	246 (93)				
Missing	2	2						
Are you in any of the following high-risk categories for severe COVID-19 infection? Severe obesity (body mass index ≥40)								
Yes	40 (4)	10 (2)	18 (6)	12 (5)	0.055			
No	943 (96)	405 (98)	285 (94)	253 (95)				
Missing	1	1						
Are you in any of the following high-risk categories for severe COVID-19 infection? Diabetes								
Yes	42 (4)	14 (3)	13 (4)	15 (6)	0.356			
No	941 (96)	401 (97)	290 (96)	250 (94)				
Missing	1	1						
Are you in any of the following high-risk categories for severe COVID-19 infection? Chronic kidney disease and dialysis								
Yes	9 (1)	1 (<1)	5 (2)	3 (1)	0.134			
No	974 (99)	414 (100)	298 (98)	262 (99)				
Missing	1	1						
Are you in any of the following high-risk categories for severe COVID-19 infection? Liver disease								
Yes	9 (1)	4 (1)	1 (<1)	4 (2)	0.335			
No	974 (99)	411 (99)	302 (100)	261 (98)				
Missing	1	1						
Are you in any of the following high-risk categories for severe COVID-19 infection? Hypertension (high blood pressure)								
Yes	118 (12)	49 (12)	31 (10)	38 (14)	0.320			
No	864 (88)	365 (88)	272 (90)	227 (86)				
Missing	2	2						

D, dental workers; ND, non–dental health care workers; NH, non–health care workers.

Table 3 describes the frequency of preventive habits for COVID-19 in daily life. All groups reported a very high frequency (>95%) of always/frequently engaging in preventive measures, such as maintaining a social distance ≥6 ft and wearing a mask in public.

**Table 3.** Preventive Habits for COVID-19 in Daily Life.

	Respondents, *n* (%)				*P* Value			
Question: Response	All	D	ND	NH	Overall	D vs. ND	D vs. NH	ND vs. NH
In the last month, have you maintained a social distance of 6 ft between you and others when going out in public?								
Never	1 (<1)	1 (<1)			<.001	0.314	<.001	0.046
Rarely	9 (1)	5 (1)	2 (1)	2 (1)				
Sometimes	43 (4)	20 (5)	15 (5)	8 (3)				
Frequently	489 (50)	227 (55)	151 (50)	111 (42)				
Always	441 (45)	162 (39)	135 (45)	144 (54)				
Missing	1	1						
In the last month, how often have you left your home to go to work, grocery store, pharmacy, doctor or other public places?								
Never	8 (1)	3 (1)	1 (<1)	4 (2)	<.001	0.014	0.043	<.001
Once every 2 wk	158 (16)	66 (16)	38 (13)	54 (20)				
1 to 2 times per week	429 (44)	184 (44)	119 (39)	126 (48)				
3 to 4 times per week	191 (19)	86 (21)	62 (20)	43 (16)				
5 to 6 times per week	133 (14)	52 (13)	56 (18)	25 (9)				
Every day	64 (7)	24 (6)	27 (9)	13 (5)				
Missing	1	1						
In the last month, how often did you wear a mask or scarf over your mouth and nose when going out in public?								
Never	2 (<1)	1 (<1)	1 (<1)		0.001	0.060	0.001	0.324
Rarely	11 (1)	6 (1)	3 (1)	2 (1)				
Sometimes	35 (4)	20 (5)	10 (3)	5 (2)				
Frequently	251 (26)	124 (30)	72 (24)	55 (21)				
Always	684 (70)	264 (64)	217 (72)	203 (77)				
Missing	1	1						

D, dental workers; ND, non–dental health care workers; NH, non–health care workers.

**Figure 1.**
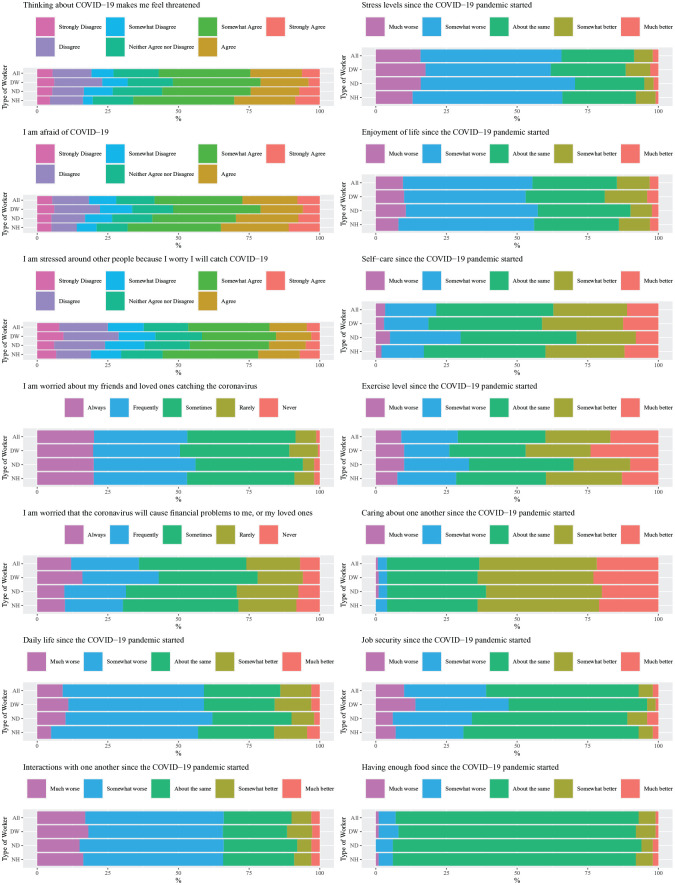
Perceived threat of COVID-19 on life experiences. Statistically significant differences (*P* < 0.05) were found for DW versus ND for the following: “I am afraid of COVID-19,” “I am worried that the coronavirus will cause financial problems to me, or my loved ones,” “Change in self-care,” “Change in exercise levels,” and “Change in job security since the COVID-19 pandemic started.” Statistically significant differences (*P* < 0.05) were found for DW versus NH for the following: “Thinking about COVID-19 makes me feel threatened,” “I am afraid of COVID-19,” “I am stressed around other people because I worry I’ll catch COVID-19,” “I am worried that the coronavirus will cause financial problems to me, or my loved ones,” and “Change in job security since the COVID-19 pandemic started.” Statistically significant differences (*P* < 0.05) were found for ND versus NH for “Change in self-care since the COVID-19 pandemic started.” DW, dental workers; ND, non–dental health care workers; NH, non–health care workers.

**Figure 2.**
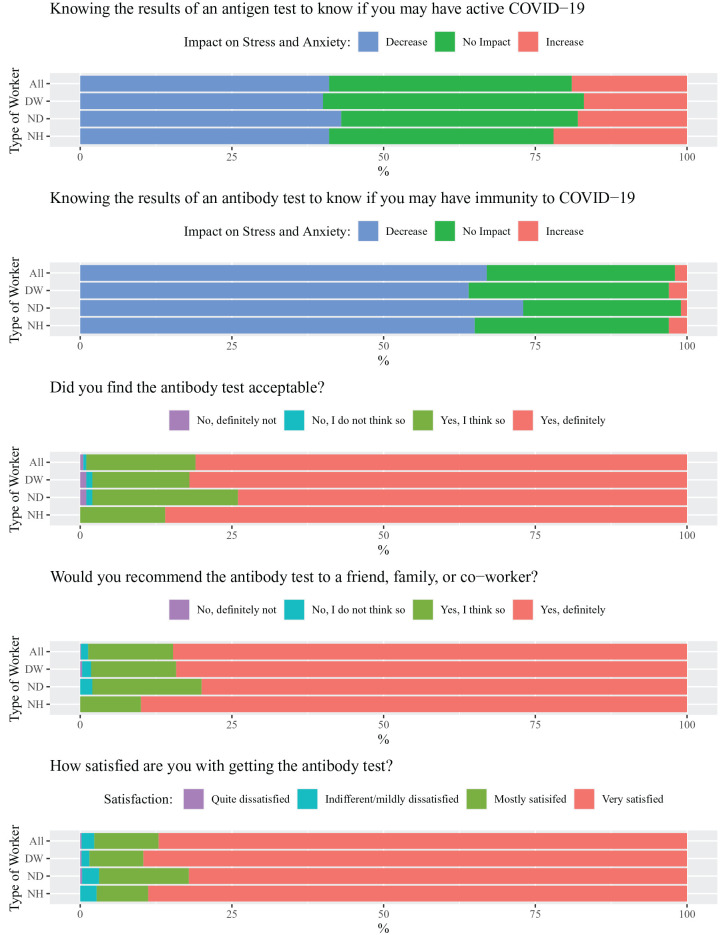
Impact of antibody test in stress and anxiety and acceptability of the test. Statistically significant differences (*P* < 0.05) were found 1) between DW and ND for stress and anxiety impact of the antibody test results and for satisfaction with the antibody test results and 2) between ND and NH for recommending the antibody test. DW, dental workers; ND, non–dental health care workers; NH, non–health care workers.

Figure 1 describes the perceived threat of COVID-19 on life experiences and aspects related to quality of life and well-being. Fifty-six percent of respondents somewhat agreed, agreed, or strongly agreed that thinking about COVID-19 made them feel threatened (score, 3.6 ± 1.6; scale, 1 = strongly agree to 7 = strongly disagree). D and ND providers were not significantly different from each other, although NH providers were more likely to feel threatened than D. Fifty-eight percent of respondents somewhat agreed, agreed, or strongly agreed that they were afraid of COVID-19 (score, 3.6 ± 1.6). D providers were less likely to be afraid than ND and NH providers. Forty-seven percent of respondents somewhat agreed, agreed, or strongly agreed that they felt stressed being around other people because of worrying of catching COVID-19 (score, 4.0 ± 1.7). D and ND providers were not significantly different from each other, although again NH providers were more likely to feel stressed than D. Over 90% of respondents were sometimes, frequently, or always worried about their friends and loved ones catching the coronavirus (score, 3.6 ± 0.9; scale, 1 = never to 5 = always), with no significant difference among the groups. More than 70% of respondents were sometimes, frequently, or always worried that COVID-19 would cause financial problems to them or their loved ones (score, 3.2 ± 1.1), with D group members less likely to be worried than ND and NH. However, as presented in Figure 2, for 67% of respondents, knowing the results of a POC antibody test would decrease their level of stress and anxiety (score, 2.6 ± 0.5; scale, 1 = increase stress and anxiety to 3 = decrease stress and anxiety), as would knowing the results of an antigen test for 41% of respondents (score, 2.2 ± 0.7).

There were no significant differences among the 3 groups regarding the impact of COVID-19 on daily life, interactions with others, stress, enjoyment of life, caring for one another, and having enough food (Fig. 1). In general, the mean scores (scale, 1 = much better to 5 = much worse) ranged between “about the same” and “somewhat worse” for the impact of the pandemic on daily life, interactions with one another, stress levels, and enjoyment of life. The mean scores also ranged between “about the same” and “somewhat worse” for job security, with significant differences among the 3 groups. However, the mean scores ranged between “about the same” and “somewhat better” for impacts on caring about one another, self-care, and exercise levels. All 3 groups reported no change in having enough food as a result of the pandemic.

Only 1% of participants (*n* = 6, 3 each in D and ND, *P* > 0.05) reported being positive for IgG antibodies and 2% for IgM antibodies (*n* = 15, 6 in D and 9 in ND, *P* > 0.05). This could reflect a low incidence of infection in this college community or could be related to the accuracy of the test, which was not the focus of this study. However, 81% of all participants found a POC test received in a dental setting “definitely” acceptable, with significantly higher responses for NH (86%) than ND (74%; Fig. 2), with 99% of participants very or mostly satisfied. Eighty-five percent of participants would “definitely” recommend a POC antibody test to a friend, family member, or coworker, with significantly higher responses for NH (90%) than ND (80%). Ninety-eight percent of participants were mostly or very satisfied with getting the antibody test in a dental setting (score, 1.2 ± 0.4; scale, 1 = very satisfied to 4 = quite dissatisfied).

## Discussion

### COVID-19 and Michigan Essential Workers

Michigan reported its first cases on March 10, with its first death on March 18. The state’s stay-at-home order was issued March 23. Cases escalated rapidly in April, with deaths peaking in late April. However, since May, cases and deaths have been decreasing. As of July 1, the state had 5,951 deaths and 64,132 confirmed cases (Michigan Department of Health and Human Services 2020), with Wayne County (city of Detroit) being the hardest hit with >21,932 cases and 2,602 deaths, while Washtenaw county (Ann Arbor, home of the University of Michigan) had 1,493 cases and 105 deaths. Participants in this study represented a generally healthy group, with few reporting being part of a health-related high-risk group for COVID-19. Participants also reported a high frequency of always or frequently engaging in preventive measures. This may explain the low number of respondents who reported having had COVID-19 symptoms within the previous 30 d. This is in contrast with a study from northern Italy, an area intensely affected by the pandemic, where 14% of dentists stated they had experienced ≥1 symptoms or signs associated with COVID-19, with the sense of fatigue and fever being the most common (8% and 7%, respectively) and with breathing difficulties and conjunctivitis being less frequent (2% each; Cagetti et al. 2020).

Possibly associated with the lower prevalence of the disease in the community, the participants’ good general health, and their adherence to preventive practices, in our study 58% thought that they had a ≤25% chance of having immunity/antibodies to COVID-19; yet, an additional 30% thought that their chance was between 26% and 50%. ND workers believed that their chance was significantly higher than the other groups, but they were also significantly more likely than the others to have continued working, to have direct contact with people and patients who were likely to have COVID-19, and to report significantly more frequent exposure to the general public since the beginning of the pandemic.

### Impact of COVID-19 on Life Experiences

The COVID-19 pandemic is having a huge psychological impact on individuals worldwide (Jakovljevic et al. 2020), and based on previous pandemics, the negative impact is likely significant for health care providers, particularly if they are frontline workers (Talevi et al. 2020). In fact, providing psychological first aid has been suggested as an essential care component for populations during and after emergencies and disasters (Talevi et al. 2020). Evidence suggests that the impact is related not only to the pandemic itself but also to the restrictive measures to contain the disease, such as quarantine, isolation, and social distancing (Brooks et al. 2020; Talevi et al. 2020), and the resulting economic consequences. Furthermore, COVID-19 has significantly affected dental care worldwide. In most countries, elective dental procedures came to a halt with the start of the pandemic. In March, the CDC (2020c) recommended that dentists prioritize emergency visits, with the goal of protecting staff and preserving personal protective equipment. According to Nasseh and Vujicic (2020), in April more than three-fourths of US dental practices were seeing emergency patients only, and another 18% were closed. This was exacerbated by the fact that the risk of cross-infection in dentistry is considered high because of aerosols produced during routine dental treatments (Szymańska 2007; Cagetti et al. 2020), similar to other coronavirus diseases (Yu et al. 2004). In fact, 65% of dentists surveyed in northern Italy during the current pandemic considered dentistry a profession at risk (Cagetti et al. 2020). However, as the pandemic continues to evolve, US dental practices have started to provide nonemergency dental care under a new framework for delivery of care (CDC 2020b), with Michigan reopening on May 29. Yet, modeling studies have predicted that US dental care spending could decline by up to 38% in 2020 and 20% in 2021 (Nasseh and Vujicic 2020). This likely contributes to the burden and stress that dental personnel are experiencing.

During the SARS coronavirus pandemic (severe acute respiratory syndrome), fear, nervousness, sadness, guilt, confusion, anger, numbness, and anxiety-induced insomnia were reported in response to the quarantine (Reynolds et al. 2008), with health care workers having higher levels of stress and psychological distress than non–health care workers (Lee et al. 2007). Moreover, health care workers exhibited more avoidance behaviors after quarantine, reported greater income loss, were consistently more affected psychologically, and were more likely to think that they had SARS and to be concerned about infecting others (Reynolds et al. 2008). Frontline health care workers were also reported to experience stress, depression, and stigma during the MERS pandemic (Middle East respiratory syndrome; Um et al. 2017). Several cross-sectional studies, mostly from China (Lai et al. 2020; Cao et al. 2020; J.Z. Huang et al. 2020; Kang et al. 2020; Li et al. 2020), as well as literature reviews (Talevi et al. 2020), have reported on the psychological well-being of health care workers during COVID-19. Findings are consistent with previous pandemics, showing that health care workers have been exposed to high levels of stressful events and expressed substantial negative mental health outcomes, such as stress-related symptoms (27% to 72%), depression (50%), anxiety (23% to 45%), and insomnia (34%; Talevi et al. 2020). Nurses, women, those of younger age, and frontline health care workers have reported more severe degrees of mental health symptoms than other groups (Lai et al. 2019; Rossi et al. 2020). Most studies surveying dentists have focused on the impact of coronaviruses on the knowledge, attitudes, and practices of dental practitioners (Brug et al. 2004; Khader et al. 2020). Our study agrees with other studies that the sampled health care workers, including dental and nondental providers, are a vulnerable group to the stresses associated with COVID-19. In our study, the majority of respondents were sometimes, frequently, or always worried about their friends and loved ones catching the coronavirus and that COVID-19 would result in financial problems. For dental workers, this may have been exacerbated by the fact that they were significantly more likely than the other groups to be unemployed or no longer working, likely influenced by the stay-at-home order and the initial CDC (2020c) recommendation to delay elective dental visits. Interestingly, even when participants were informed of the need for more evidence to understand the value of POC tests, the majority still decided to be tested and responded that knowing the results of a POC antibody or antigen test would decrease the level of stress and anxiety. For all 3 groups, there was a slight negative impact of the pandemic on aspects related to daily life, interactions with others, stress levels, enjoyment of life, and job security. However, there was also a slight positive impact on aspects likely associated with spending more time at home, such as caring about one another, self-care, and exercise.

### Role of Dentists and Acceptability of Tests by Dentists

The growing body of evidence on the connections of oral and general health continues to drive the need for expanding collaborations among dental and other health care workers to improve health (Fontana et al. 2017). In fact, dentistry has the potential to play a role within the broader health care system, during and beyond the current pandemic. Because dental professionals, unlike other health care workers, see patients who are otherwise generally well, they are uniquely positioned to help with surveillance efforts of community health. For example, in situations such as the current pandemic, the dental workforce can help provide timely testing and triage to other sections of the health care system (Holden et al. 2020). In fact, an important application of serologic tests is to understand antibody responses to the SARS-CoV-2 infection and vaccination. Currently, it is unclear whether there is a different antibody response associated with asymptomatic cases versus those presenting with varied severity of symptoms and how long the antibody responses last. It is also unclear whether antibody titers correlate with virus neutralization and protection from reinfection. These questions are important as we determine differences between antibody responses generated by natural infection and eventual vaccination (Krammer and Simon 2020). Detection of protective immune responses are also important considerations for health care workers. Serologic tests can further inform the prevalence of infection in different populations, with POC tests being easy to escalate. In addition, as information about which test or combination of tests may be most accurate and adequate to use, dentists can be part of the health care workforce that aids in data collection and triage to other members of the health care team for testing and/or treatment. Furthermore, interprofessional education and collaborative care have become a priority in dental curriculum reform. Including competencies associated with community surveillance and triage efforts, as well as infection control, will enhance the opportunities for interprofessional practice (Fontana et al. 2017). In our study, the majority of participants were definitely satisfied with a POC test received in a dental setting and would definitely recommend it to others. Also, the pilot-testing program in a dental school setting demonstrated that dental personnel are a willing workforce that can be mobilized during medical emergencies to provide services such as testing. Our survey showed that dental workers were significantly more likely than the other 2 groups to use personal protective equipment at work prior to COVID-19, as they have long been attentive to employing procedures to safely provide care given the high-risk environments in which they work. This may also explain why dental providers reported being less afraid of COVID-19 than the other 2 groups. At the start, testing was done under the direction of an oral surgeon with a medical license, but during the course of the study, the Michigan state licensing board gave authority for dentists to conduct medical screening and testing.

Our study shares limitations with many of the existing questionnaire studies previously discussed in that it is cross-sectional and uses self-reported questions (some with limited validation) on a limited convenient sample. To encourage comparisons across samples and facilitate data integration and collaboration, survey items related to COVID-19 started to be made available in online survey platform repositories. We adapted many of our questions, when available, on items available in these repositories. In addition, other factors not included in this study could have affected the life experiences of essential workers (e.g., socioeconomic status, personality, ongoing psychological disorders) and the interpretation of the data. All of these limitations affect the generalizability of our findings regarding the psychological well-being of essential workers during COVID-19 in the population studied in Michigan. To better understand these data, future studies relying on qualitative methods could provide important additional details on these experiences.

In conclusion, regardless of these limitations, our findings agree with others in the literature that dental providers are as vulnerable as other essential workers to the threats and psychological impacts of COVID-19. Our findings also suggest that testing may alleviate stress and anxiety, and they support the acceptability and satisfaction of testing done in dental settings.

## Author Contributions

M. Fontana, L. McCauley, M. Fitzgerald, R. Eber, contributed to conception, design, data acquisition, analysis, and interpretation, drafted and critically revised the manuscript; G.J. Eckert, contributed to design, data analysis, and interpretation, drafted and critically revised the manuscript; E. Yanca, contributed to design, data acquisition, and interpretation, drafted and critically revised the manuscript. All authors gave final approval and agree to be accountable for all aspects of the work.

## References

[bibr1-2380084420962399] Brooks SK Webster RK Smith LE Woodland L Wessely S Greenberg N Rubin GJ 2020 The psychological impact of quarantine and how to reduce it: rapid review of the evidence. Lancet. 395(10227):912–920. 32112714 10.1016/S0140-6736(20)30460-8 PMC7158942

[bibr2-2380084420962399] Brug J Aro AR Oenema A de Zwart O Richardus JH Bishop GD 2004 SARS risk perception, knowledge, precautions, and information sources, the Netherlands. Emerg Infect Dis. 10(8):1486–1489. 15496256 10.3201/eid1008.040283 PMC3320399

[bibr3-2380084420962399] Cagetti MG Cairoli JL Senna A Campus G. 2020 COVID-19 outbreak in North Italy: an overview on dentistry. A questionnaire survey. Int J Environ Res Public Health. 17(11):E3835. 32481672 10.3390/ijerph17113835 PMC7312000

[bibr4-2380084420962399] Cao J Wei J Zhu H Duan Y Geng W Hong X Jiang J Zhao X Zhu B. 2020 A study of basic needs and psychological wellbeing of medical workers in the fever clinic of a tertiary general hospital in Beijing during the COVID-19 outbreak. Psychother Psychosom. 89(4):252–254. 32224612 10.1159/000507453 PMC7179543

[bibr5-2380084420962399] Centers for Disease Control and Prevention. 2020a. Coronavirus disease 2019 (COVID-19) [accessed 2020 4 1]. https://www.cdc.gov/coronavirus/2019-ncov/faq.html#covid19-basics

[bibr6-2380084420962399] Centers for Disease Control and Prevention. 2020b. Framework for healthcare systems providing non-COVID-19 clinical care during the COVID-19 pandemic [accessed 2020 Jun 1]. https://www.cdc.gov/coronavirus/2019-ncov/hcp/framework-non-COVID-care.html

[bibr7-2380084420962399] Centers for Disease Control and Prevention. 2020c. Guidance for dental settings: interim infection prevention and control guidance for dental settings during the COVID-19 response [accessed 2020 Jun 1]. https://www.cdc.gov/coronavirus/2019-ncov/hcp/dental-settings.html#Background

[bibr8-2380084420962399] Fontana M Gonzalez-Cabezas C de Peralta T Johnsen D. 2017 Dental education required for the changing healthcare environment. J Dent Educ. 81(8):eS153–eS161. 10.21815/JDE.017.022 28765467

[bibr9-2380084420962399] Guan W-J Ni Z-Y Hu Y Liang W-H Ou C-Q He J-X Liu L Shan H Lei C-L Hui DS, et al 2020 Clinical characteristics of coronavirus disease 2019 in China. N Engl J Med. 382(18):1708–1720. 32109013 10.1056/NEJMoa2002032 PMC7092819

[bibr10-2380084420962399] Guo L Ren L Yang S Xiao M Chang D Yang F Cruz CSD Wang Y Wu C Xiao Y, et al 2020 Profiling early humoral response to diagnose novel coronavirus disease (COVID-19). Clin Infect Dis. 71(15):778–785. 32198501 10.1093/cid/ciaa310 PMC7184472

[bibr11-2380084420962399] Holden ACL Shaban ARZ Spallek H 2020 COVID-19 and the dental profession: professional tensions and ethical quandaries. A COVID-19 Sydney policy paper in depth. Sydney (Australia): University of Sydney [accessed 2020 Jun 17]. https://www.sydney.edu.au/content/dam/corporate/documents/sydney-policy-lab/policy-paper_covid-19–and-the-dental-profession.pdf

[bibr12-2380084420962399] Huang C Wang Y Li X Ren L Zhao J Hu Y Zhang L Fan G Xu J Gu X, et al 2020 Clinical features of patients infected with 2019 novel coronavirus in Wuhan, China. Lancet. 395(10223):497–506. 31986264 10.1016/S0140-6736(20)30183-5 PMC7159299

[bibr13-2380084420962399] Huang JZ Han MF Luo TD Ren AK Zhou XP 2020 Mental health survey of medical staff in a tertiary infectious disease hospital for COVID-19. Zhonghua Lao Dong Wei Sheng Zhi Ye Bing Za Zhi. 38(3):192–195. 32131151 10.3760/cma.j.cn121094-20200219-00063

[bibr14-2380084420962399] Jakovljevic M Bjedov S Jaksic N Jakovljevic I. 2020 COVID-19 pandemia and public and global mental health from the perspective of global health security. Psychiatr Danub. 32(1):6–14. 32303023 10.24869/psyd.2020.6

[bibr15-2380084420962399] Kang L Ma S Chen M Yang J Wang Y Li R Yao L Bai H Cai Z Yang BX, et al 2020 Impact on mental health and perceptions of psychological care among medical and nursing staff in Wuhan during the 2019 novel coronavirus disease outbreak: a cross-sectional study. Brain Behav Immun. 87:11–17. 32240764 10.1016/j.bbi.2020.03.028 PMC7118532

[bibr16-2380084420962399] Khader Y Al Nsour M Al-Batayneh OB Saadeh R Bashier H Alfaqih M Al-Azzam S Al-Shurman BA 2020 Dentists’ awareness, perception, and attitude regarding COVID-19 and infection control: a cross-sectional study among Jordanian dentists. JMIR Public Health Surveill. 6(2):e18798. 32250959 10.2196/18798 PMC7147327

[bibr17-2380084420962399] Krammer F Simon V. 2020 Serology assays to manage COVID-19. Science. 368(6495):1060–1061. 32414781 10.1126/science.abc1227

[bibr18-2380084420962399] Lai J Ma S Wang Y Cai Z Hu J Wei N Wu J Du H Chen T Li R, et al 2020 Factors associated with mental health outcomes among health care workers exposed to coronavirus disease 2019 JAMA Netw Open. 3(3):e203976. 32202646 10.1001/jamanetworkopen.2020.3976 PMC7090843

[bibr19-2380084420962399] Lee AM Wong JG McAlonan GM Cheung V Cheung C Sham PC Chu C-M Wong P-C Tsang KWT Chua SE 2007 Stress and psychological distress among SARS survivors 1 year after the outbreak. Can J Psychiatry. 52(4):233–240. 17500304 10.1177/070674370705200405

[bibr20-2380084420962399] Lerner AM Eisinger RW Lowy DR Petersen LR Humes R Hepburn M Cassetti MC 2020 COVID-19 serology studies workshop: meeting report. Immunity [accessed 2020 6 23]. doi:10.1016/j.immuni.2020.06.012 PMC7309808 32610080

[bibr21-2380084420962399] Li Z Ge J Yang M Feng J Qiao M Jiang R Bi J Zhan G Xu X Wang L, et al 2020 Vicarious traumatization in the general public, members, and non-members of medical teams aiding in COVID-19 control. Brain Behav Immun. 88:916–919. 32169498 10.1016/j.bbi.2020.03.007 PMC7102670

[bibr22-2380084420962399] Mao L Jin H Wang M Hu Y Chen S He Q Chang J Hong C Zhou Y Wang D, et al 2020 Neurological manifestations of hospitalized patients with coronavirus disease 2019 in Wuhan, China. JAMA Neurol. 77(6):683–690. 32275288 10.1001/jamaneurol.2020.1127 PMC7149362

[bibr23-2380084420962399] Michigan Department of Health and Human Services. 2020 Coronavirus [accessed 2020 Jul 1]. https://www.michigan.gov/coronavirus/

[bibr24-2380084420962399] Nasseh K Vujicic M. 2020 Modeling the impact of COVID-19 on US dental spending—June 2020 update. Health Policy Institute Research Brief. Chicago (IL): American Dental Association [accessed 2020 6 20]. https://www.ada.org/~/media/ADA/Science%20and%20Research/HPI/Files/HPIBrief_0620_1.pdf?&utm_source=adaupdate&utm_medium=email&utm_content=cv-hpi-june-2020-research-brief&utm_campaign=covid-19

[bibr25-2380084420962399] National Institutes of Health. 2020 Repository of COVID-19 research tools [accessed 2020 4 20]. https://dr2.nlm.nih.gov/

[bibr26-2380084420962399] PhenX Toolkit. 2020 COVID-19 protocol [accessed 2020 4 20]. https://www.phenxtoolkit.org/covid19

[bibr27-2380084420962399] Reynolds DL Garay JR Deamond SL Moran MK Gold W Styra R. 2008 Understanding, compliance and psychological impact of the SARS quarantine experience. Epidemiol Infect. 136(7):997–1007. 17662167 10.1017/S0950268807009156 PMC2870884

[bibr28-2380084420962399] Rossi R Socci V Pacitti F Di Lorenzo G Di Marco A Siracusano A Rossi A. 2020 Mental health outcomes among front and second line health workers associated with the COVID-19 pandemic in Italy. medRxiv [accessed 2020 9 9]. https://www.medrxiv.org/content/10.1101/2020.04.16.20067801v1

[bibr29-2380084420962399] Szymańska J 2007 Dental bioaerosol as an occupational hazard in a dentist’s workplace. Ann Agric Environ Med. 14(2):203–207. 18247451

[bibr30-2380084420962399] Talevi D Socci V Carai M Carnaghi G Faleri S Trebbi E di Bernardo A Capelli F Pacitti F. 2020 Mental health outcomes of the COVID-19 pandemic. Riv Psichiatr. 55(3):137–144. 32489190 10.1708/3382.33569

[bibr31-2380084420962399] Tu Y-F Chien C-S Yarmishyn AA Lin Y-Y Luo Y-H Lin Y-T Lai W-Y Yang D-M Chou S-J Yang Y-P, et al 2020 Review of SARS-CoV-2 and the ongoing clinical trials. Int J Mol Sci. 21(7):2657. 10.3390/ijms21072657 PMC7177898 32290293

[bibr32-2380084420962399] Um DH Kim JS Lee HW Lee SH 2017 Psychological effects on medical doctors from the Middle East respiratory syndrome (MERS) outbreak: a comparison of whether they worked at the MERS occurred in hospital or not, and whether they participated in MERS diagnosis and treatment. J Korean Neuropsychiatr Assoc. 56(1):28–34.

[bibr33-2380084420962399] World Health Organization. 2020 Mental health considerations during COVID-19 outbreak. Geneva (Switzerland): World Health Organization [accessed 2020 Jun 17]. https://www.who.int/docs/default-source/coronaviruse/mental-health-considerations.pdf?sfvrsn=6d3578af_2

[bibr34-2380084420962399] Yu IT Li Y Wong TW Tam W Chan AT Lee JH Leung DY Ho T. 2004 Evidence of airborne transmission of the severe acute respiratory syndrome virus. N Engl J Med. 350(17):1731–1739. 15102999 10.1056/NEJMoa032867

